# Single-Cell Multiomic Approaches Reveal Diverse Labeling of the Nervous System by Common Cre-Drivers

**DOI:** 10.3389/fncel.2021.648570

**Published:** 2021-04-14

**Authors:** Rachel A. Keuls, Ronald J. Parchem

**Affiliations:** ^1^Development, Disease Models & Therapeutics Graduate Program, Baylor College of Medicine, Houston, TX, United States; ^2^Center for Cell and Gene Therapy, Stem Cells and Regenerative Medicine Center, Baylor College of Medicine, Houston, TX, United States; ^3^Department of Molecular and Cellular Biology, Baylor College of Medicine, Houston, TX, United States; ^4^Department of Neuroscience, Baylor College of Medicine, Houston, TX, United States

**Keywords:** single-cell mRNA sequencing, single-cell ATAC sequencing, cranial neural crest, neural tube, Wnt1-Cre2, Sox10-Cre, cell diversity

## Abstract

Neural crest development involves a series of dynamic, carefully coordinated events that result in human disease when not properly orchestrated. Cranial neural crest cells acquire unique multipotent developmental potential upon specification to generate a broad variety of cell types. Studies of early mammalian neural crest and nervous system development often use the Cre-loxP system to lineage trace and mark cells for further investigation. Here, we carefully profile the activity of two common neural crest Cre-drivers at the end of neurulation in mice. RNA sequencing of labeled cells at E9.5 reveals that Wnt1-Cre2 marks cells with neuronal characteristics consistent with neuroepithelial expression, whereas Sox10-Cre predominantly labels the migratory neural crest. We used single-cell mRNA and single-cell ATAC sequencing to profile the expression of *Wnt1* and *Sox10* and identify transcription factors that may regulate the expression of Wnt1-Cre2 in the neuroepithelium and Sox10-Cre in the migratory neural crest. Our data identify cellular heterogeneity during cranial neural crest development and identify specific populations labeled by two Cre-drivers in the developing nervous system.

## Introduction

Neural crest cells are a unique population of multipotent progenitors that have the developmental potential to give rise to a variety of diverse cell types, including peripheral neurons, glia, cranial bone and cartilage, and melanocytes. Neural crest cells arise during a very dynamic stage of early development at the end of gastrulation, beginning with neural plate border formation, followed by the specification of neural crest progenitors, epithelial-to-mesenchymal transition (EMT), migration, and finally, terminal differentiation. The dynamic developmental trajectory of the neural crest has contributed to the difficulty of studying these cells *in vivo*.

Early studies in chicken, frog, and fish uncovered key gene regulatory networks (GRNs) and core transcription factor families important for the sequential stages of neural crest development. Several transcription factor families are conserved among neural crest specification genes, such as *Pax3/7*, *AP2*, and *SoxE* factors (e.g., *Sox9* and *Sox10*). Mutations in these factors cause severe, life-threatening disorders that usually manifest in infants and young children, such as neuroblastoma (Pasterls et al., 1993; Shakhova et al., 2012; Shirley et al., 2012; Weiss et al., 2012), skeletal dysplasia (campomelic dysplasia), and abnormalities of the jaw and palate, which compromise the airways at birth (Houston et al., 1983; Shinwell et al., 1988; Kwok et al., 1995; Mansour et al., 1995; Sock et al., 2003; Herman and Siegel, 2012).

Despite the severe phenotypes that are characteristic of neural crest-related defects, far less is known about mammalian neural crest development compared to the avian, fish, and amphibian models used in the foundational studies that constitute much of the current knowledge of neural crest development. The extent to which mechanisms uncovered using the classical models translate to mammalian models is unclear. Indeed, the neural crest-specific ablation of factors identified in other animal models as being critical for EMT did not affect the early stages of mouse neural crest development (Brault et al., 2001; Hari et al., 2002; Jia et al., 2007; Büchmann-Møller et al., 2009). It is not clear whether this failure to identify genes critical to early neural crest development reflects true species-specific differences or technical limitations of the available tools to study neural crest (Barriga et al., 2015).

Two of the most commonly used Cre-drivers for neural crest studies are Wnt1-Cre (Danielian et al., 1998; Lewis et al., 2013) and Sox10-Cre (Stine et al., 2009). The utility of Sox10-Cre is that it has a high specificity for labeling migratory neural crest cells and neural crest-derived structures. The Sox10-Cre transgenic mouse strain, designated as the S4F:Cre mouse, contains a *Sox10* distal enhancer MCS4 and a *c-Fos* minimal promoter followed by the Cre-recombinase coding sequence. At E9.5, Sox10-Cre induced recombination in the migratory neural crest, the otic placode, and the dorsal root ganglia of the pharyngeal arches, with little evidence of recombination in the dorsal neural tube (Stine et al., 2009). While the specificity to migratory neural crest is a great advantage to this Cre-driver, one drawback is that Sox10-Cre cannot be used for studies of the premigratory neural crest.

Wnt1-Cre is a well-established Cre-driver with over 50 citations that are frequently used to study multiple aspects of early brain and premigratory neural crest development in mice (Danielian et al., 1998; The Jackson Laboratory, 2020). The Wnt1-Cre transgene contains a Wnt1 promoter followed by the Cre-recombinase coding region, the Wnt1 coding region, and the 3’ Wnt1 enhancer. This transgenic mouse line is accepted as a robust way to label approximately 96% of neural crest cells, including both premigratory and migratory cells (Hari et al., 2012). However, Wnt1-Cre has been reported to be expressed in cell types other than the neural crest, including cells of the midbrain, which results in ectopic activation of Wnt signaling and disrupted midbrain development. This led to the development of the Wnt1-Cre2 transgene, which labels neural crest similarly to the first version of Wnt1-Cre (Lewis et al., 2013) without ectopic activation of Wnt signaling. The Wnt1-Cre2 transgene consists of the Wnt1 promoter region followed by the Cre-recombinase coding region and the 3’ Wnt1 enhancer. Thus, Wnt1-Cre2 lacks the coding sequence for Wnt1 to avoid ectopic activation of Wnt signaling. However, the recombination achieved with Wnt1-Cre2 has not been thoroughly investigated using single-cell transcriptomic approaches and the importance to do so increases as more studies use Wnt1-Cre2 to harvest early neural crest cells (Lumb et al., 2017; Soldatov et al., 2019).

In the present study, we carefully profile the recombination of the ROSA26^Tomato/+^ Cre-reporter (Ai9; Luche et al., 2007) achieved *in vivo* with both Wnt1-Cre2 (Lewis et al., 2013) and Sox10-Cre (Stine et al., 2009) in the cranial region of mouse embryos at E9.5. In addition to efficient labeling of neural crest, we reveal expression of both Wnt1-Cre2 and Sox10-Cre in other cell types and therefore identify differences in the transcriptomes of cells harvested using either Cre. To study endogenous expression, we used single-cell transcriptomics to demonstrate that *Wnt1* and *Sox10* are expressed in different subpopulations of the neural crest at E9.5. Furthermore, we use single-cell ATAC sequencing to analyze chromatin structure and identify accessible motifs containing predicted binding sites for transcription factors, which may regulate the expression of *Wnt1* and *Sox10* in the neural tube and neural crest. Our combined *in vivo* and multiomics approach reveals the coordination of gene expression and chromatin accessibility contributing to the cellular diversity of early central and peripheral nervous system development.

## Materials and Methods

### Immunofluorescence

Mouse embryos were dissected in phosphate-buffered saline, pH 7.4 (PBS), fixed in 3.7% formaldehyde overnight at 4°C, and washed in PBS containing 0.1% Triton (PT). Embryos were stored at −30°C in methanol and were rehydrated at the time of use in PT. Embryos were cryopreserved using a sucrose gradient of 10%, 20% then 30% w/v sucrose in PT, followed by 1:1 30% sucrose:OCT Compound (Fisher Scientific 23730571) and 100% OCT. Embryos were flash-frozen in OCT for cryosectioning using dry ice and 100% ethanol bath and were stored at −80°C until sectioning. Cryo-sectioning was performed at 10 um and slides were stored at −80°C until staining. Sections on slides were washed with PT and blocking was performed at room temperature for 1 h in 5% Gibco normal goat serum (16210064) and 1% bovine serum albumin (Fisher Scientific BP1600100). Primary antibodies were diluted in blocking solution and applied to tissue overnight at 4°C [Sox9 (EMD Millipore AB5535; 1:1,000) Pax3 (DSHB PAX3; 1:100)]. Secondary antibodies (AlexaFluor) diluted in blocking buffer (1:500) were applied for 1.5 h at room temperature. Sections were mounted with Fluoromount G (Fisher Scientific OB10001). Images of the cross-sections were taken on Zeiss LSM780 or LSM 980. Wholemount embryos were imaged on Leica M165FC dissecting microscope with a Leica DFC 3000G camera or Zeiss LSM780.

### Embryo Dissociation and Cell Sorting

Wnt1-Cre2 (Lewis et al., 2013) or Sox10-Cre (Stine et al., 2009) mice were crossed with ROSA26^Tomato/+^ mice (Luche et al., 2007) to lineage trace neural crest cells at E9.5. Mouse embryos were decapitated anterior to the otic placode. The cranial region was enzymatically dissociated with papain at room temperature combined with gentle pipetting until a single-cell suspension was achieved. An equal volume of FBS was used to quench the enzyme. The single-cell suspension was filtered, spun at 300 g for 5 min, and resuspended in PBS with 1% BSA. Samples were filtered and fluorescent cells were sorted on a BD FACSAria III instrument with a 70-μm nozzle into a 1.5 ml Eppendorf tube that contained Trizol-LS (Thermo Fisher Scientific, 10296028).

### Bulk RNA Isolation, Library Preparation, and Sequencing

Total RNA was extracted from cells using RNeasy Micro Kit (QIAGEN 74004). For mRNA sequencing cDNA synthesis was performed using SMART-Seq Ultra Low Input RNA Kit for Sequencing (Takara 634889) from approximately 500 pg of total RNA. cDNA was validated using the High Sensitivity NGS Fragment Analysis Kit (Agilent formerly AATI DNF-474-0500) on a 12-Capillary Fragment Analyzer. Quantification was determined using the Quant-iT dsDNA Assay Kit, high sensitivity (Thermo Fisher Q33120), and 100 pg of cDNA was tagmented and ligated using the Nextera XT DNA Library Kit (Illumina FC-131-1024) at $\frac{1}{2}$ volumes to produce sequencing libraries. The resulting libraries were validated using the High Sensitivity NGS Fragment Analysis Kit on a 12-Capillary Fragment Analyzer and quantified using the Quant-iT dsDNA Assay Kit, high sensitivity. Equal concentrations of libraries were pooled, denatured, diluted, and subjected to paired-end sequencing using the Mid Output v2.5 kit (Illumina FC-404-2001) on a NextSeq550 following the manufacturer’s instructions.

### Bulk RNA-Sequencing Bioinformatic Analysis

Sequencing files from each flow cell lane were downloaded and the resulting FASTQ files were merged. Quality control was performed using fastQC (v0.10.1). Reads were mapped to the mouse genome mm10 assembly using STAR (v2.5.0a). In R (v3.5.2), gene count matrices were built with Bioconductor packages Rsamtools (v2.0.0) and GenomicFeatures (v1.32.2). mRNA-sequencing datasets were annotated with UCSC transcripts downloaded from Illumina iGenomes in GTF file format. We determined reads per million (RPM) using GenomicAlignments (v1.16.0). Principal component analysis (PCA) was performed using an rlog transformed gene expression matrix of global gene expression >1 for each region. DESeq2 (v1.20.0) was used for differential gene expression analysis and read count normalization. Expression heat maps were generated using ComplexHeatmap (v2.0.0). Biological Process GO analyses were determined using Enrichr and visualized using ggplot.

### Single-Cell Sample Preparation and Bioinformatic Analysis

Wildtype mouse embryos were decapitated at the otic placode and the cranial region was enzymatically dissociated with papain at room temperature combined with gentle pipetting until a single-cell suspension was achieved. At which time the dissociation was quenched with an equal volume of FBS. The single-cell suspension was filtered (Falcon 352235), spun at 300 g for 5 min, and resuspended in DMEM with 10% FBS. For single-cell ATAC sequencing, nuclei were isolated by resuspending cell pellet in lysis buffer (10 mM Tris-Cl pH 7.4, 10 mM NaCl, 3 mM MgCl_2_, 0.1% Tween-20, 0.01% Nonidet P40, 0.01% Digitonin, 1% BSA). Lysis was diluted with wash buffer (10 mM Tris-Cl pH 7.4, 10 mM NaCl, 3 mM MgCl_2_, 0.1% Tween-20, 1% BSA, 0.1% Tween-20) and nuclei were pelleted by spinning at 500 g for 5 min. Nuclei were then washed, pelleted, and resuspended in nuclei buffer (10× Genomics 2000153). Nuclei count and quality were assessed on a hemocytometer using 0.4% Trypan blue to stain nuclei. GEM generation was performed on a 10× Chromium Controller Instrument on Chromium Next GEM Chip G for mRNA (10× Genomics 1000120) and Next GEM Chromium Chip H for ATAC (10× Genomics 1000161). Libraries were subsequently prepped using Chromium Next GEM Single Cell 3’ Library Kit v3.1 for mRNA (10× Genomics 1000158) and Chromium Next GEM Single Cell ATAC Library Kit v1.1 for ATAC (10× Genomics 1000163). Chromium Single Index Kit T Set A (10× Genomics 1000213) was used to index mRNA libraries and i7 Multiplex Kit N Set A (10× Genomics 1000084) was used to index ATAC libraries. Libraries were validated using the High Sensitivity NGS Fragment Analysis Kit on a 12-Capillary Fragment Analyzer and quantified using the Quant-iT dsDNA Assay Kit, high sensitivity. Equal concentrations of libraries were pooled, denatured, diluted, and subjected to paired-end sequencing using the Mid Output v2.5 kit (Illumina FC-404-2001) on a NextSeq550 following the manufacturer’s instructions. At E9.5 16,949, cells were sequenced from a C57BL/6 wildtype mouse embryo (128, 243, 041 reads; an average of 7,566 reads per cell). Single-cell mRNA Raw bcl files were downloaded using Illumina’s BaseSpaceCLI version 0.10.7 and were converted to fastq files using 10× Genomics Cell Ranger mkfastq and were aligned to the mm10 genome using 10× Genomics Cell Ranger count. Data were subsequently log normalized and clustered using five statistically significant principal components in Seurat version 3.1.5 (Butler et al., 2018). Mitochondrial genes and cells with <200 UMI and >2,500 UMIs were filtered out. Cell populations were identified for single-cell mRNA data as in previous studies (Pijuan-Sala et al., 2019) and cell markers are provided in Supplementary Table 1. Single-cell ATAC raw bcl files were downloaded using Illumina’s BaseSpaceCLI version 0.10.7 and were converted to fastq files using 10× Genomics Cell Ranger ATAC mkfastq (version 1.1.0) and were aligned to the mm10 genome using 10× Genomics Cell Ranger ATAC count. Peaks were unified, quantified, and visualized in Seurat and Signac (version 0.2). Single-cell ATAC data was overlayed with cell populations from annotated single-cell mRNA similar to previous studies (Stuart et al., 2019, 2020 preprint and motifs were called within 2 kb of the loci of interest by Homer. Enrichr was used for gene ontology analyses.

### RNAScope

RNAScope was performed using the Multiplex Fluorescent Reagent Kit v2 (ACD Bio 323100). Embryos were dissected, fixed, cryopreserved, and cryosectioned as above. Slides were stored at −80°C until use and were brought to room temperature. Tissue sections were re-fixed for 15 min at room temperature and subsequently dehydrated in 50% ethanol, followed by 70% and 100% ethanol. Hydrogen peroxide was applied to tissue sections for 10 min at room temperature and antigen retrieval was performed for 5 min at >99°C in a Black and Decker HS800 steamer. Sections were subsequently washed in distilled water, dehydrated in 100% ethanol, dried at room temperature, and subsequently treated with RNAScope Protease III for 30 min at 40°C. Mm-Wnt1-C2 RNAScope 2.5 LS Probe (ACD Bio 4011098-C2) was diluted 1:10 with Mm-Sox10 RNAScope 2.5 LS Probe (ACD Bio 435938) and the probe mixture was hybridized at 40°C for 2 h. Signal was amplified with RNAScope Multiplex FL v2 AMP1-3 for 30 min each at 40°C. Sox10 signal was developed by incubating tissue sections for 15 min at 40°C in RNAScope Multiplex FL v2 hP-C1 and Opal 570 [Perkin Elmer FP1488A (diluted 1:2,000 in TSA buffer)] was applied for 30 min at 40°C. HRP blocker was applied for 15 min at 40°C and Wnt1 signal was subsequently developed by incubating tissue sections for 15 min at 40°C in RNAScope Multiplex FL v2 hP-C2 and Opal 690 [Perkin Elmer FP1497A (diluted 1:500 in TSA buffer)] was applied for 30 min at 40°C. Images were taken on a Zeiss LSM 980.

### Animal Work

All research and animal care procedures were approved by the Baylor College of Medicine Institutional Animal Care and Use Committee and housed in the Association for Assessment and Accreditation of Laboratory Animal Care-approved animal facility at Baylor College of Medicine. All strains were maintained on the C57BL/6 background. Adult genotyping was performed by lysing 1–2 mm ear clippings in 75 μl 25 mM NaOH 0.2 mM EDTA at 98°C for 1 h and neutralized with 75 μl 40 mM Tris-Cl, pH 5.5. Embryo genotyping was performed by digesting yolk sac tissue overnight in lysis buffer [50 mM Tris-HCl (pH 8.0), 10 mM EDTA, 100 mM NaCl, 0.1% SDS, and 5 mg/ml proteinase K]. Cell debris was removed and an equal amount of isopropanol was used to precipitate DNA at −30°C for 1 h. DNA was pelleted by a 30 min centrifugation, washed with 70% ethanol, and resuspended in water. PCR for all alleles was performed using 40 cycles 95 °C for 20 s and touch-down annealing at 64°C, 62°C, 60°C, 58°C, followed by 40 s extension at 72°C. All PCR primers and expected band sizes are in Table 1.

**Table 1:** PCR primers and expected band sizes.

Genotyping primer	Sequence	Expected band sizes
Tomato forward	CACTTGCTCTCCCAAAGTCG	550 bp wildtype and 300 bp mutant
Wildtype reverse	TAGTCTAACTCGCGACACTG	
Tomato reverse	GTTATGTAACGCGGAACTCC	
Cre 26	CCT GGA AAA TGC TTC TGT CCG	300 bp Gabra control and 400 bp Cre
Cre 36	CAG GGT GTT ATA AGC AAT CCC	
Gabra 12	CAA TGG TAG GCT CAC TCT GGG AGA TGA TA	
Gabra 70	AAC ACA CAC TGG CAG GAC TGG CTA GG	

### Results

#### Wnt1-Cre2 and Sox10-Cre Label the Neural Crest and the Neural Tube

**Figure 1:**
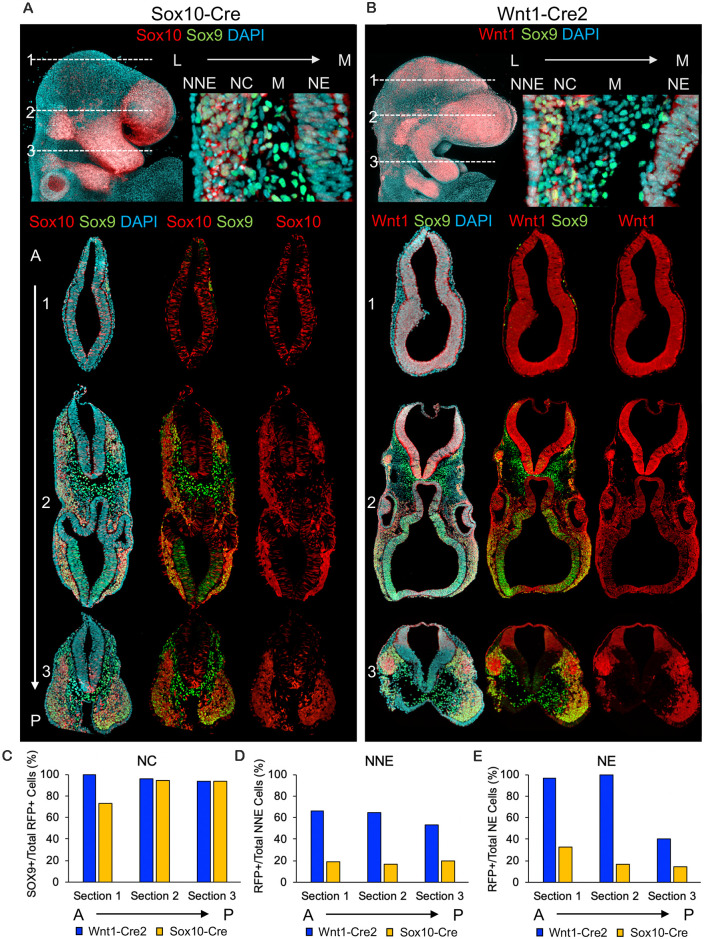
Wnt1-Cre2 and Sox10-Cre label neural crest and cells of the neural tube. **(A)** Wholemount and sequential transverse cross-sections of embryos in which ROSA26^Tomato/+^ embryos were recombined using Sox10-Cre and **(B)** Wnt1-Cre. Axial positions of transverse cross-sections are noted for each Cre-driver. **(C)** Quantification of neural crest, **(D)** non-neural ectoderm and **(E)** neuroepithelial labeling obtained from Sox10-Cre and Wnt1-Cre.

To compare the expression of the most common Cre-drivers used to label neural crest, we crossed both Wnt1-Cre2 (Lewis et al., 2013) and Sox10-Cre (Stine et al., 2009) with ROSA26^Tomato/+^ [Gt(ROSA)26Sor^tm9(CAG-tdTomato)Hz^; Luche et al., 2007] and harvested embryos at E9.5, the time at which neural crest cells are delaminating and migrating into cranial structures such as the frontonasal process and pharyngeal arches (Supplementary Figure 1A). Analysis of wholemount embryos revealed that recombination from Wnt1-Cre2 was more widespread throughout the neural tube as compared to Sox10-Cre, which was largely localized to the neural crest in the frontonasal process and pharyngeal arches (Figures 1A,B, Supplementary Figure 1A).

To more carefully analyze and compare the cell populations labeled from each Cre-driver, we used immunofluorescence (IF) for the neural crest marker SOX9 in transverse cross-sections of the cranial region in E9.5 embryos labeled with Sox10-Cre or Wnt1-Cre2. Recombination with Sox10-Cre resulted in robust neural crest labeling (Figure 1A). Interestingly, we found Tomato-positive cells scattered within the anterior neural tube and to a lesser extent in more posterior sections, where the labeled cells were more restricted to the ventral neuroepithelium (Figure 1A). Labeling with Wnt1-Cre2 revealed nearly ubiquitous Tomato expression within SOX9-positive neural crest as well as throughout the neuroepithelium just anterior to the mid/hindbrain boundary including a low level of scattered labeling within the neuroepithelium of the forebrain (Figure 1B). When we analyzed the co-localization of the SOX9-positive neural crest, we found that both Cre-drivers labeled greater than 90% of SOX9-positive cells in each section (Figure 1C). We found that 50–60% of the non-neural ectoderm within each section was Tomato-positive using Wnt1-Cre2 whereas less than 20% of the non-neural ectoderm per section was Tomato-positive using Sox10-Cre, consistent with a lateral source of Wnt signaling during neural plate border formation (Figure 1D). In each section, Wnt1-Cre2 labeled 100% of neuroepithelial cells anterior to the mid/hindbrain boundary while Sox10-Cre labeled less than 30% of neural tube cells in each section (Figure 1E).

Although recombination by Wnt1-Cre2 in the neuroepithelium allows for the labeling of premigratory neural crest, it also limits the ability to isolate a pure population of neural crest cells. This also applies to studies using Wnt1-Cre2 to delete a gene of interest within the neural crest, as the use of Wnt1-Cre2 would cause deletion in the mid-and hindbrain region. These results are in agreement with previous findings that Wnt1-Cre2 labels cell types other than neural crest. For example, recombination has been observed in the pharyngeal arch and frontonasal process epithelial cells (Lewis et al., 2013). Analysis of wholemount embryos from Lewis et al. (2013) also suggests recombination in neuroepithelial cells anterior to the mid/hindbrain boundary. Additionally, we observed some scattered recombination driven by Sox10-Cre in the anterior neural tube, suggesting Sox10-Cre also marks cell types other than migratory neural crest.

#### Cells Harvested Using Wnt1-Cre2 Have a Neuroepithelial Gene Signature Compared to Cells Harvested Using Sox10-Cre

**Figure 2:**
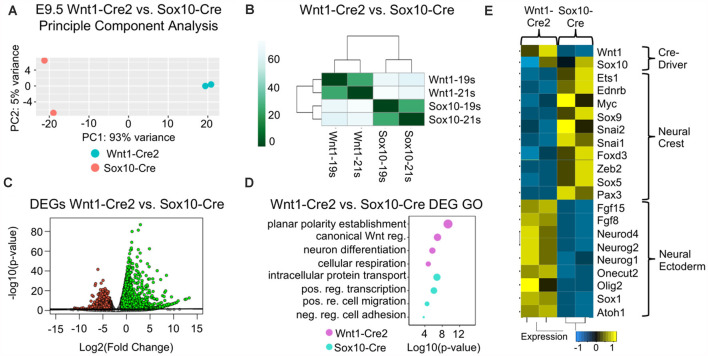
Cells harvested using Wnt1-Cre2 have a neuroepithelial gene signature compared to cells harvested using Sox10-Cre. **(A)** Principal component analysis (PCA) plot and **(B)** heatmap normalized counts per million reads (CPM) showing that samples group together by Cre-driver. **(C)** Volcano plot of the significantly up- and downregulated genes obtained from comparing Wnt1-Cre2 to Sox10-Cre. **(D)** Gene ontology (GO) analysis of the significant differentially expressed genes. Categories were ordered by −log10(*p*-value) and dot size represents −log10(*p*-value). **(E)** Heatmap of normalized CPM values for neural crest and neuronal genes from cells harvested with Wnt1-Cre2 or Sox10-Cre.

To identify changes in gene expression that may result from the differential labeling of cells between Wnt1-Cre2 and Sox10-Cre, we crossed both Wnt1-Cre2 and Sox10-Cre with ROSA26^Tomato/+^. Tomato-positive cells were isolated from the cranial region using fluorescence-activated cell sorting (FACS), and we analyzed their transcriptomes using mRNA sequencing (Supplementary Figures 2A,B). A principal component analysis was used to compare samples harvested with each Cre-driver and found that samples clustered together based on the Cre-driver used to harvest them (Figures 1A,B). Differential expression analysis was used to compare transcriptomes of cells harvested with Wnt1-Cre2 to cells harvested with Sox10-Cre. We found more upregulated genes as compared to downregulated genes, consistent with a broader labeling when using Wnt1-Cre2 as compared to Sox10-Cre (Figure 2C). We predicted that genes upregulated in our comparison were those enriched in Wnt1-Cre2 that confer a more neuroepithelial identity while the downregulated genes are enriched in Sox10-Cre and would provide a more migratory neural crest-like identity. Indeed, gene ontology (GO) analysis of the upregulated genes enriched in cells harvested with Wnt1-Cre2 revealed predicted functions such as neuronal differentiation and planar cell polarity, consistent with neuroepithelial labeling, as these cells will go on to differentiate into neurons of the central nervous system. GO analysis of the downregulated genes revealed predicted function in the positive regulation of cell migration and negative regulation of cell adhesion consistent with a migratory neural crest identity (Figure 2D). The expression of neural crest specification genes such as *Sox9*, *Pax3*, and *Ets1* was greater in cells harvested at E9.5 using Sox10-Cre as compared to those harvested using Wnt1-Cre2. Similarly, expression of EMT genes such as *FoxD3*, *Snai2*, and *Sox5* was more enriched in cells harvested at E9.5 using Sox10-Cre. Neuronal genes such as *Atoh1*, *Fgf15*, and *Olig2*, were more highly expressed in cells labeled by Wnt1-Cre2 (Figure 2E). These results are consistent with Wnt1-Cre2 broadly labeling cells of the neural tube near the mid-hindbrain boundary while the labeling achieved with Sox10-Cre is more restricted to migratory neural crest.

#### Single-Cell Sequencing Reveals Little Co-expression of Wnt1 and Sox10 in E9.5 Neural Crest Cells

**Figure 3:**
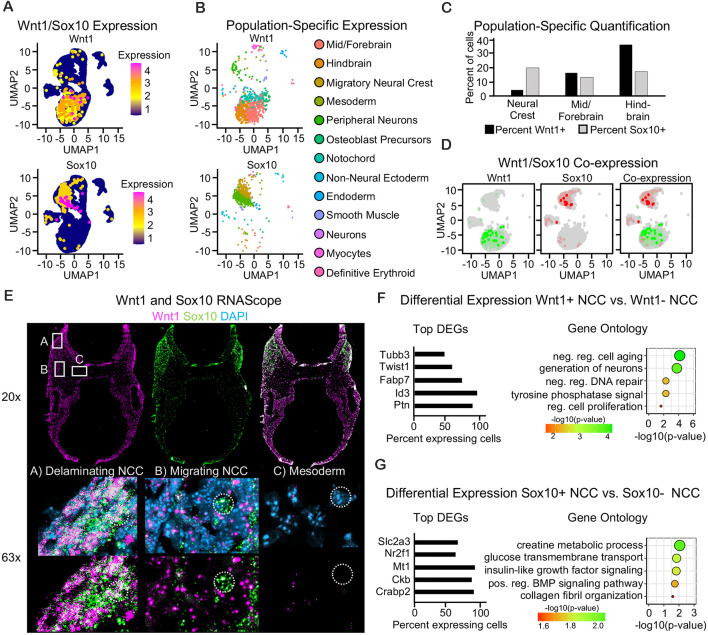
Single-cell sequencing reveals little co-expression of Wnt1 and Sox10 in E9.5 neural crest cells. **(A)** UMAP plot showing expression of *Wnt1* and *Sox10* in cells of the E9.5 cranial region. **(B)** UMAP plot with *Wnt1*- and *Sox10*-expressing cells colored by cell population. **(C)** Bar plot of the percent of cells within the neural tube and neural crest expressing *Wnt1* or *Sox10*. **(D)** Blended UMAP plot showing little co-expression of *Wnt1* and *Sox10*. **(E)** Images of RNAScope for *Sox10* and *Wnt1* in E9.5 transverse cross-section through the cranial region showing co-localization in the delaminating neural crest, minimal co-localization in the migratory neural crest, and minimal expression of both *Wnt1* and *Sox10* in cells of the mesoderm. The location of the 63× image is noted within the 20× image. **(F)** Bar plot of differentially expressed genes and GO analysis of *Wnt1*-positive cells and **(G)** *Sox10*-positive cells. Categories were ordered by −log10(*p*-value) and dot size represents −log10(*p*-value).

To further interrogate cell populations expressing *Wnt1* and *Sox10*, we used single-cell mRNA sequencing of the cranial region from an E9.5 mouse embryo (Supplementary Figures 3A–D). When we mapped the expression of *Wnt1*, we found that the majority of *Wnt1*-positive cells were found in the mid-, fore- and hindbrain (Figures 3A,B). The majority of *Sox10*-positive cells were found in migratory neural crest and peripheral neuron populations (Figures 3A,B). Indeed, Sox10-Cre has been shown to label the dorsal root ganglia (Stine et al., 2009). Similar to our results from lineage tracing, quantification of the number and percent of cells in each population of the neuroepithelium and the neural crest revealed that *Wnt1* is expressed in more cells of the mid/forebrain and the hindbrain neuroepithelium as compared to *Sox10* at E9.5. Indeed, *Sox10* was expressed in more neural crest cells as compared to *Wnt1* (Figure 3C). When we interrogated the co-expression of *Sox10* and *Wnt1* in the neural crest, interestingly, we found very few co-expressing cells (Figure 3D). Fluorescent RNAScope for *Wnt1* and *Sox10* confirmed the expression of *Wnt1* in both the neural tube and neural crest and expression of *Sox10* specifically in the neural crest. Similar to single-cell sequencing, RNAScope similarly revealed minimal co-expression of *Wnt1* and *Sox10*, except in delaminating neural crest cells. Delaminating neural crest immediately adjacent to the neural tube expressed both *Wnt1* and *Sox10* consistent with homogeneity of neural crest undergoing EMT (Figure 3E). Differential expression analysis was used to identify the genes enriched in the *Wnt1*-expressing neural crest as compared to the *Sox10*-expressing neural crest and found each population had a unique gene signature (Figures 3F,G). Genes enriched in *Wnt1*-positive neural crest were involved in neuron formation, regulation of cell proliferation, and DNA repair regulation (Figure 3F). Genes enriched in *Sox10*-positive neural crest were involved in glucose transport, BMP signaling, and collagen fibril organization (Figure 3G). Taken together, these findings support a distinct gene signature of *Wnt1*- and *Sox10*-expressing neural crest at E9.5.

#### Single-Cell ATAC Sequencing Reveals Accessible Motifs Near Wnt1 in the Neuroepithelium and Sox10 in the Neural Crest

**Figure 4:**
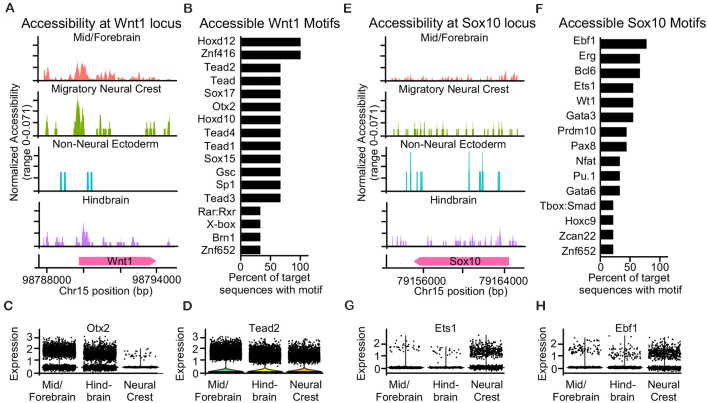
Single-cell ATAC sequencing reveals accessible motifs near Wnt1 in the neuroepithelium and Sox10 in neural crest. **(A)** Coverage plot of accessibility at the *Wnt1* locus in ectoderm-derived cell populations. **(B)** Bar plot of accessible motifs at the *Wnt1* locus. **(C)** Dot plot showing expression of *Otx2* and **(D)** *Tead2* transcription factors with accessible predicted motifs at the *Wnt1* locus. **(E)** Coverage plot of accessibility at the *Sox10* locus in ectoderm-derived cell populations. **(F)** Bar plot of accessible motifs at the *Sox10* locus. **(G)** Dot plot showing expression of *Ets1* and **(H)** *Ebf1* transcription factors with accessible predicted motifs at the *Sox10* locus.

Since we found distinct gene signatures of *Wnt1*- and *Sox10*-expressing neural crest and broad labeling of the neuroepithelium when we used Wnt1-Cre2, we performed single-cell ATAC sequencing of the cranial region from an E9.5 mouse embryo to identify and compare accessible transcription factor motifs near the transcriptional start sites at the *Wnt1* and *Sox10* gene loci. We hypothesized that accessible regions of chromatin near the *Wnt1* locus may contain motifs for factors expressed in both the neural tube and neural crest while accessible regions of chromatin near the *Sox10* locus may contain motifs for factors expressed specifically in the migratory neural crest. Chromatin structure around the *Wnt1* locus was largely accessible in cells of the mid/forebrain, possibly contributing to the labeling of the neuroepithelium that we observed with Wnt1-Cre2 (Figure 4A). In the migratory neural crest and the hindbrain, chromatin at the *Wnt1* locus is accessible at the transcriptional start site as well as specific regions upstream. Accessible regions upstream of the *Wnt1* locus are similar between the migratory neural crest and the hindbrain which may suggest that these regulatory regions enable the labeling of both the premigratory and migratory neural crest. Chromatin structure around the *Wnt1* locus in cells of the non-neural ectoderm was largely closed. However, we found that some cells of the non-neural ectoderm were labeled when we crossed Wnt1-Cre2 with ROSA26^Tomato/+^. These findings suggest that cells of the non-neural ectoderm may transiently express *Wnt1* earlier in development, possibly during the division of the non-neural, neural, and neural plate border domains of the epithelium (Figure 4A). Motifs present in the accessible regions near the *Wnt1* locus included *Hoxd12*, *Tead2*, *Sox17*, *Znf416*, and *Otx2* (Figure 4B, Supplementary Figure 4A). Of these transcription factors, we found that *Tead2* was robustly expressed in both the neural crest and neuroepithelium and *Otx2* was more highly expressed in the neuroepithelium compared to the neural crest (Figures 4C,D). Indeed, *Otx2* has been suggested as a specification factor in premigratory neural crest (Finkelstein and Perrimon, 1991; LeDouarin et al., 1999; Williams et al., 2019), and our results suggest that *Otx2* expression may decline in the late migratory neural crest. These findings suggest that broad expression of *Wnt1* could be due to transcriptional activation by transcription factors expressed in both the neural crest and neural tube.

We found the chromatin structure around *Sox10* was slightly less accessible as compared to *Wnt1* at E9.5 (Figures 4A,E). Chromatin around the *Sox10* locus was largely inaccessible in the non-neural ectoderm and was accessible similarly within the migratory neural crest, mid/forebrain, and hindbrain (Figure 4E). The accessible regions of chromatin around the *Sox10* locus contained motifs for transcription factors such as *Ebf1, Erg, Bcl6, Ets1*, and* Wt1* (Figures 4F, Supplementary Figure 4B). Of the transcription factors predicted to bind at the accessible regions near the *Sox10* locus, we found that *Ebf1* and *Ets1* were most highly expressed and enriched in the neural crest with little expression in either the mid/forebrain or hindbrain populations (Figures 4G,H). Our analysis of motifs present in the accessible regions of chromatin upstream of *Wnt1* reveals transcriptional control by factors expressed in both the neuroepithelium and neural crest, while motifs present in the accessible regions of chromatin upstream of *Sox10* reveal transcriptional control by factors expressed exclusively in the neural crest.

#### Single-Cell Sequencing Reveals Little Co-expression of Sox9 and Pax3 in E9.5 Neural Crest Cells

**Figure 5:**
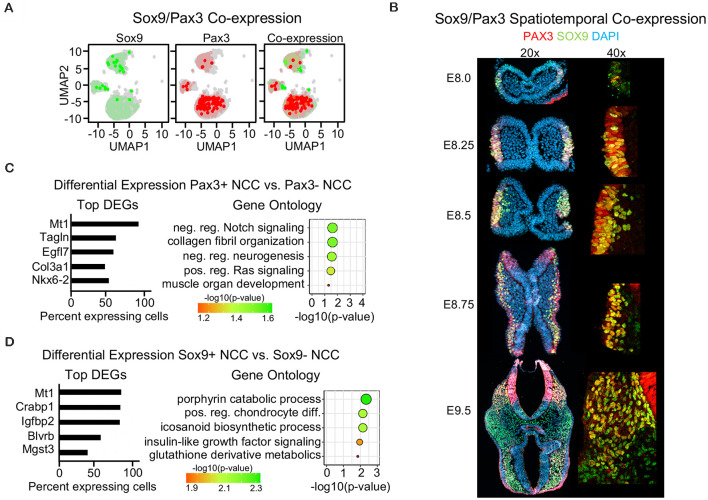
Single-cell sequencing reveals little co-expression of Sox9 and Pax3 in E9.5 neural crest cells. **(A)** Blended UMAP plot showing little co-expression of *Sox9* and *Pax3*. **(B)** Immunofluorescence of transverse mouse embryo sections for PAX3 and SOX9 from E8.0 to E9.5. **(C)** Bar plot of differentially expressed genes and GO analysis of *Pax3*-positive cells and **(D)** *Sox9*-positive cells at E9.5.

Since we found that *Wnt1* and *Sox10* were largely not co-expressed within neural crest cells at E9.5, we analyzed co-expression of canonical neural crest markers *Pax3* and *Sox9*. Surprisingly, we found that *Pax3* and *Sox9* were similarly not co-expressed at E9.5 (Figure 5A, Supplementary Figures 5A,B). Moreover, IF staining for PAX3 and SOX9 during neural crest development revealed little co-expression at the earliest stages of formation between E8.0 to E8.25, suggesting that early neural crests are heterogeneous in mice (Figure 4B, Supplementary Figure 4B). In migratory neural crest at E8.5 and E8.75, the co-expression of PAX3 and SOX9 increased, consistent with previous studies (Soldatov et al., 2019). However, at E9.5, PAX3 and SOX9 are largely not co-expressed except for a few delaminating crests located near the dorsal neural tube consistent with the divergence of neural crest populations as they begin to commit to a terminal fate (Figure 5B). Differential expression between the *Pax3*- or *Sox9*-positive neural crest and non-expressing cells revealed a unique gene signature (Figures 5C,D). Genes enriched in *Pax3*-positive neural crest are involved in Ras and Notch signaling and neural crest terminal differentiation (Figure 5C). Genes enriched in *Sox9*-positive neural crest are involved in chondrocyte differentiation as well as porphyrin, icosanoid, and glutathione metabolic processes (Figure 5D). However, we did identify *Mt1* as a gene enriched in both *Pax3*-positive and *Sox9*-positive neural crest. *Mt1* is an antioxidant gene activated by the redox sensing transcription factor *Nrf1* (Ohtsuji et al., 2008). Differential expression analyses suggest that *Pax3*- and *Sox9*-positive cells may differ in their terminal differentiation capacity and use of specific metabolic processes, yet remain similarly enriched for genes involved in metabolism. Taken together, our findings identify different types of nervous system cells captured by commonly used Cre-drivers, identify transcription factors that may contribute to the labeling obtained in the neural tube by Wnt1-Cre2, and highlight the heterogeneity of E9.5 neural crest cells.

### Discussion

We use multiple approaches to shed light on the extent of labeling from common neural crest Cre-drivers and demonstrate the importance of carefully profiling recombination. We find using IF of transverse serial cross-sections that Wnt1-Cre2 ubiquitously labels the neural tube near the mid/hindbrain boundary at E9.5 resulting in a neuronal-like gene signature from cells isolated using FACS as compared to cells harvested with Sox10-Cre. Previously, Wnt1-Cre2 was profiled in E8.5 and E9.5 wholemount embryos and transverse section through the E9.5 pharyngeal arch to show that labeling of the neural crest with Wnt1-Cre2 was similar to that of the original Wnt1-Cre2 (Lewis et al., 2013). Our results expand on the understanding of labeling by Wnt1-Cre2 by analyzing serial cross-sections along the anterior/posterior axis at E9.5. While Wnt1-Cre2 corrects for the ectopic activation of Wnt signaling and associated phenotypes, Wnt1-Cre2 robustly labels cells of the neural tube, which may be a limitation for studies that require neural crest specificity. The labeling of the neuroepithelium by Wnt1-Cre2 and the endogenous expression of *Wnt1* in the neuroepithelium at E9.5 does allow for the continual labeling of the premigratory neural crest. However, the labeling of premigratory neural crest by Wnt1-Cre2 may not accurately target the process of neural crest formation as previous studies have found that recombination by Wnt1-Cre2 may occur too late to allow for early neural crest studies (Brault et al., 2001; Hari et al., 2002; Jia et al., 2007; Büchmann-Møller et al., 2009). Our work profiling the recombination of Wnt1-Cre2 and Sox10-Cre focuses on E9.5 neural crest populations. Future studies may be aimed at profiling the recombination of Wnt1-Cre2 during neural crest formation. It will be important to determine how Wnt1-Cre2 labels cells at the earliest stages of neural crest development. For example, it remains to be determined whether premigratory cells possess an ectomesenchymal vs. neural fate bias and whether both fates are comparably captured by Wnt1-Cre2 in mice. Furthermore, it has been shown that *Wnt1* expression declines in neural crest as they delaminate from the neural tube (Zervas et al., 2004; Kléber et al., 2005; Rabadán et al., 2016; Bhattacharya et al., 2018; Hutchins and Bronner, 2018). We similarly found robust *Wnt1* expression in neuroepithelial cells compared to neural crest and little co-expression of *Wnt1* and *Sox10* in migratory neural crest at E9.5. This may reflect the downregulation of *Wnt1* after the neural crest migrates away from the neural tube or the possibility that *Wnt1* and *Sox10* label different neural crest cell populations or derivatives.

We identified predicted motifs of transcription factors that may promote the expression of *Wnt1* in both the neural tube and neural crest. We identified predicted motifs for *Otx2* and *Tead2* in the accessible regions of chromatin around the *Wnt1* locus and both of these factors are expressed in the neural tube. *Otx2* expression parallels that of *Wnt1*; *Otx2* was expressed in more cells of the neural tube than neural crest at E9.5. Furthermore, *Otx2* has been suggested to be an early mammalian neural crest transcription factor and have a role in the neural specification (Finkelstein and Perrimon, 1991; LeDouarin et al., 1999; Williams et al., 2019). *Otx2* expression may decline in more mature neural crest cells. *Tead2* is known to interact with an enhancer element to regulate the expression of *Pax3*, a factor that is also expressed in both the neural crest and the dorsal neural tube. Similar to the labeling achieved with Wnt1-Cre2, we find *Tead2* is expressed in both neural crest cells and cells of the neural tube. We also identified predicted motifs of transcription factors that may promote the expression of *Sox10* specifically in the neural crest. We found that accessible regions of chromatin around the *Sox10* locus at E9.5 contain motifs for *Ets1* and *Ebf1*. *Ets1* is a canonical cranial neural crest specification transcription factor (Barembaum and Bronner, 2013). *Ebf1* has been identified as a neural crest migration transcription factor (Simões-Costa et al., 2014). Our multiomics approach enables the identification of accessible motifs which may promote expression of *Wnt1* in both migratory neural crest and neural tube, as well as endow specificity of *Sox10* to migratory neural crest. However, with our approach, single-cell ATAC data was obtained from one embryo and overlayed with single-cell mRNA data from a second embryo and future studies may benefit from newer technologies where both types of libraries can be constructed from the same cell.

Our findings reveal cellular heterogeneity that should be taken into account when selecting a Cre-driver for labeling the nervous system in mice. The heterogeneity of neural crest has long been a question of interest as these cells must maintain the multipotent differentiation potential to form various derivatives during migration. The degree to which migratory neural crests are a homogeneous cell population that will subsequently diverge vs. heterogeneous populations that exist immediately after delamination from the neural tube remains to be determined. Heterogeneity of neural crest cells was evident *via* immunofluorescence for canonical neural crest transcription factors SOX9 and PAX3 at most stages of neural crest development. However, we did find considerable SOX9 and PAX3 co-expression during delamination, similar to *Wnt1* and *Sox10* co-expression, and a recent study which found that neural crest cells are similar during EMT and then subsequently diverge into various lineages (Soldatov et al., 2019). Studies in chick revealed subpopulations of the neural crest that largely do not co-express neural crest transcription factors consistent with our findings (Roellig et al., 2017). Regardless of the transcription factors expressed in each subpopulation of neural crest, our analysis revealed that neural crest subpopulations are similarly metabolically active, consistent with previous studies (Bhattacharya et al., 2020; Keuls et al., 2020). Taken together, our findings uncover unique aspects of cellular diversity amongst early cranial neural crest and identify specific populations of cells targeted by Wnt1-Cre2 and Sox10-Cre during early neural development in mice.

## Data Availability Statement

The datasets presented in this study can be found in online repositories. Raw and processed single-cell mRNA and single-cell ATAC data can be found under GEO SuperSeries GSE167456. Bulk mRNA sequencing data can be found on the GEO database under GSE137227.

## Ethics Statement

The animal study was reviewed and approved by Baylor College of Medicine Institutional Animal Care and Use Committee.

## Author Contributions

RAK contributed to Figures 1–5 and Supplementary Figures 1–5, writing/editing of the manuscript, and conceptualizing the project. RJP contributed to editing the manuscript. All authors contributed to the article and approved the submitted version.

## Conflict of Interest

The authors declare that the research was conducted in the absence of any commercial or financial relationships that could be construed as a potential conflict of interest.

## Supplementary Material

SUPPLEMENTARY FIGURE 1 Wnt1-Cre2 and Sox10-Cre label the neural crest and the neural tube. Whole-mount images of Wnt1-Cre2 and Sox10-Cre were used to lineage trace neural crest.

SUPPLEMENTARY FIGURE 2 Cells harvested using Wnt1-Cre2 have a neuroepithelial gene signature compared to cells harvested using Sox10-Cre. **(A)** Violin and **(B)** density plot showing that the expression values for all samples used in the analysis are comparable.

SUPPLEMENTARY FIGURE 3 Single-cell sequencing reveals little co-expression of Wnt1 and Sox10 in E9.5 neural crest cells. **(A)** Violin plot and **(B)** scatter plot showing the count of RNA transcripts and mapped features for each cell. **(C)** Jackstraw plot showing the significance of 20 principal components. **(D)** Elbow plot showing the standard deviation of 20 principal components. **(E)** RNAScope for Wnt1 and Sox10 in transverse cranial cross-sections at 63× and corresponding co-localization scatter plot showing the minimal overlap of *Wnt1* and *Sox10*.

SUPPLEMENTARY FIGURE 4 Single-cell ATAC sequencing reveals accessible motifs near Wnt1 in the neuroepithelium and Sox10 in neural crest. **(A)** Bar plot showing the significance of predicted motifs present in the accessible regions of chromatin at the *Wnt1* and **(B)** *Sox10* loci.

SUPPLEMENTARY FIGURE 5 Single-cell sequencing reveals little co-expression of Sox9 and Pax3 in E9.5 neural crest cells. **(A)** Bar plot quantifying the co-expression of PAX3 and SOX9. **(B)** Immunofluorescence of transverse mouse embryo cross-sections for PAX3 and SOX9 from E8.0 to E9.5.
