# Supplementary figures and images for: Single-Cell Multiomic Approaches Reveal Diverse Labeling of the Nervous System by Common Cre-Drivers
Source: Front Cell Neurosci. 2021 Apr 14;15:648570. doi: 10.3389/fncel.2021.648570 (PMC8079645; doi:10.3389/fncel.2021.648570)

Sox10-Cre

Wnt1-Cre2

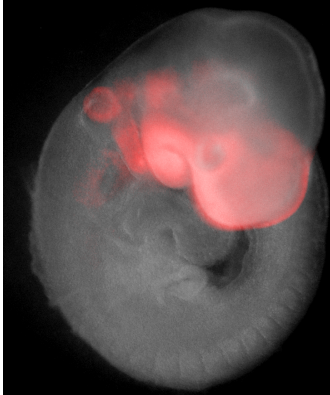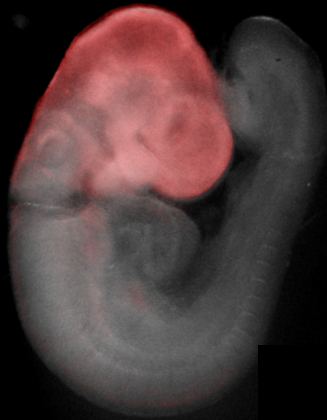

Supplement: SUPPLEMENTARY FIGURE 1 — Wnt1-Cre2 and Sox10-Cre label the neural crest and the neural tube. Whole-mount images of Wnt1-Cre2 and Sox10-Cre were used to lineage trace neural crest. [file Image_1.PDF]

**A**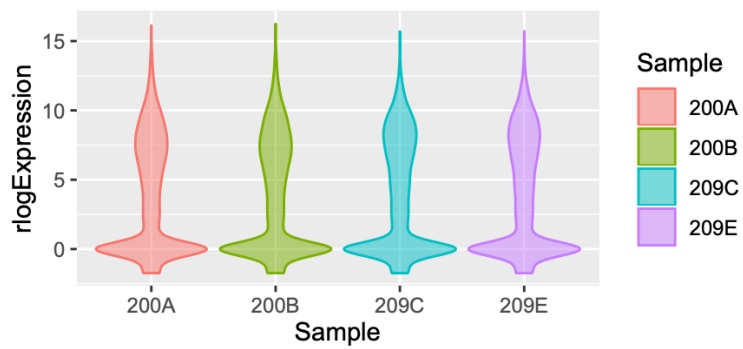**B**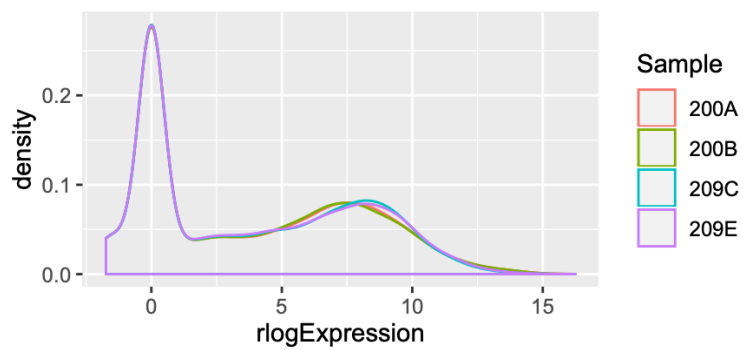

Supplement: SUPPLEMENTARY FIGURE 2 — Cells harvested using Wnt1-Cre2 have a neuroepithelial gene signature compared to cells harvested using Sox10-Cre. (A) Violin and (B) density plot showing that the expression values for all samples used in the analysis are comparable. [file Image_2.PDF]

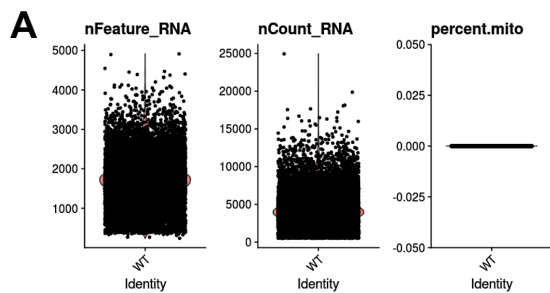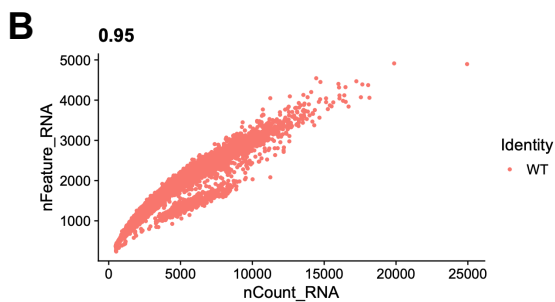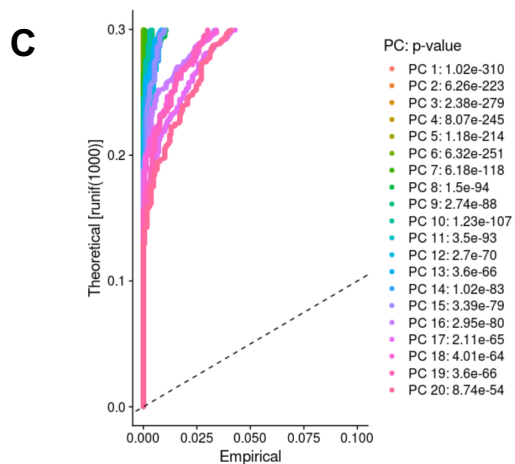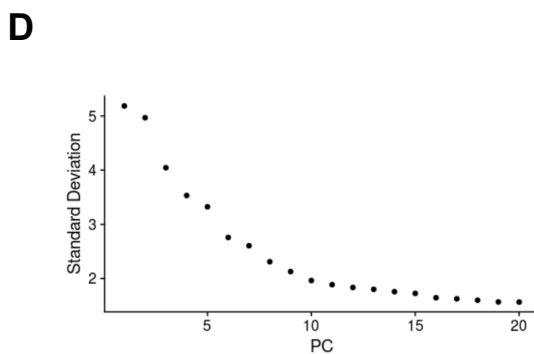

**E**

Wnt1 and Sox10 RNAScope

Wnt1 Sox10

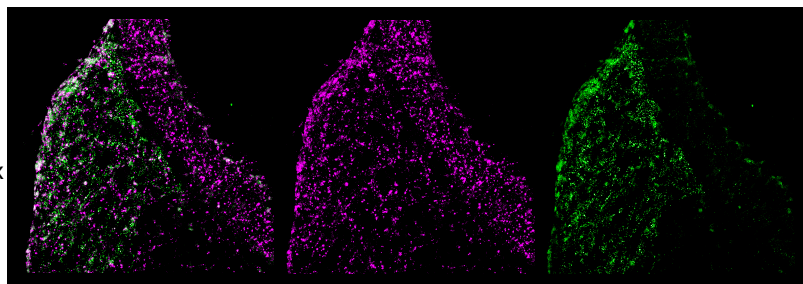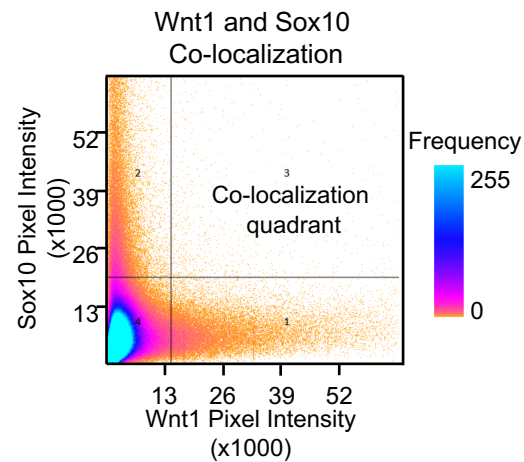

Supplement: SUPPLEMENTARY FIGURE 3 — Single-cell sequencing reveals little co-expression of Wnt1 and Sox10 in E9.5 neural crest cells. (A) Violin plot and (B) scatter plot showing the count of RNA transcripts and mapped features for each cell. (C) Jackstraw plot showing the significance of 20 principal components. (D) Elbow plot showing the standard deviation of 20 principal components. (E) RNAScope for Wnt1 and Sox10 in transverse cranial cross-sections at 63× and corresponding co-localization scatter plot showing the minimal overlap of Wnt1 and Sox10. [file Image_3.PDF]

**A**

Motifs present in open regions  
at Wnt1 locus

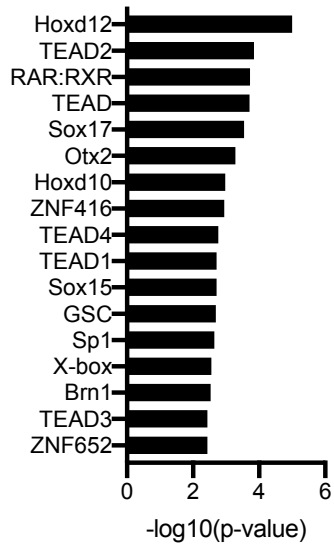**B**

Motifs present in open regions  
at Sox10 locus

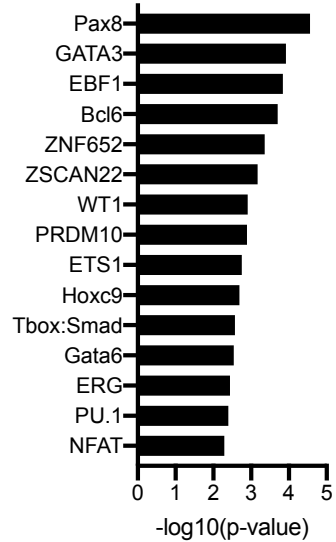

Supplement: SUPPLEMENTARY FIGURE 4 — Single-cell ATAC sequencing reveals accessible motifs near Wnt1 in the neuroepithelium and Sox10 in neural crest. (A) Bar plot showing the significance of predicted motifs present in the accessible regions of chromatin at the Wnt1 and (B) Sox10 loci. [file Image_4.PDF]

**A** E9.5 Single-cell Sox9/Pax3 Co-expression

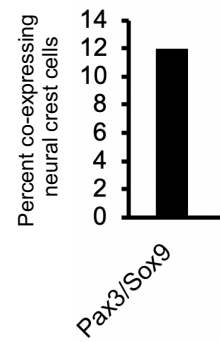

**B** Sox9/Pax3 Spatiotemporal Co-expression

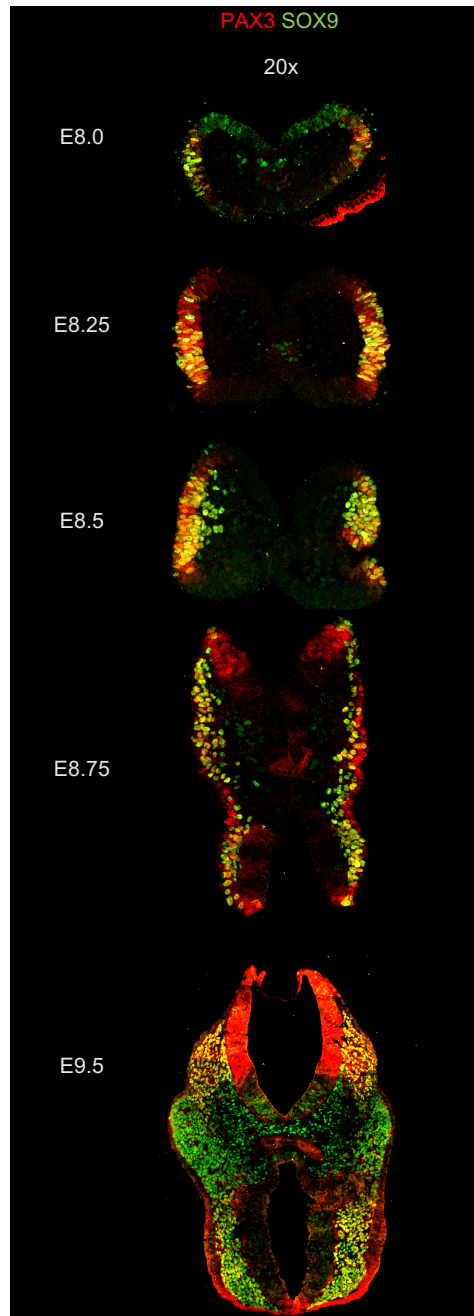

Supplement: SUPPLEMENTARY FIGURE 5 — Single-cell sequencing reveals little co-expression of Sox9 and Pax3 in E9.5 neural crest cells. (A) Bar plot quantifying the co-expression of PAX3 and SOX9. (B) Immunofluorescence of transverse mouse embryo cross-sections for PAX3 and SOX9 from E8.0 to E9.5. [file Image_5.PDF]
